# Transcutaneous Spinal Magnetic Stimulation Affects Subthalamic Activity in Parkinson's Disease

**DOI:** 10.1002/mds.70035

**Published:** 2025-09-03

**Authors:** Anna Letícia de Moraes Alves, Juliana da Silva Simões, Luiz Ricardo Trajano da Silva, Arnaldo Fim Neto, Fábio Godinho, Rafael Bernhart Carra, Janaína Reis Menezes, Glaucia Aline Nunes, Daniele Salgado Barros, Thiago Trajano da Silva, Maria Sheila Guimarães Rocha, Manoel Jacobsen Teixeira, Egberto Reis Barbosa, Diogo Coutinho Soriano, Rubens Gisbert Cury

**Affiliations:** ^1^ Movement Disorders Center, Department of Neurology, School of Medicine University of São Paulo São Paulo Brazil; ^2^ Center for Engineering, Modeling and Applied Social Sciences Federal University of ABC (UFABC) São Bernardo do Campo Brazil; ^3^ Samsung R&D Institute Brazil (SRBR), Campinas São Paulo Brazil; ^4^ Functional Neurosurgery, Department of Neurology, School of Medicine University of São Paulo São Paulo Brazil; ^5^ Department of Neurology Santa Marcelina Hospital São Paulo São Paulo Brazil; ^6^ Hospital Israelita Albert Einstein Department of Neurology Brazil

**Keywords:** Parkinson's disease, spinal cord stimulation, subthalamic nucleus activity, transcutaneous spinal magnetic stimulation, local field potential

We report the effects of transcutaneous spinal magnetic stimulation (TsMS) on subthalamic nucleus (STN) local field potential (LFP) of Parkinson's disease (PD) patients with prior STN deep brain stimulation (DBS). This study is the first to investigate whether TsMS influences STN LFP patterns in humans, thereby advancing understanding of its mechanisms. The rationale for spinal stimulation originates from studies in parkinsonian monkeys demonstrating that spinal cord stimulation (SCS) can alleviate parkinsonism by disrupting abnormal beta‐band neuronal synchrony, linked to bradykinesia in PD.^1^ Despite this evidence, clinical results of SCS and TsMS in humans remain inconsistent.^2^, ^3^ The most favorable TsMS outcomes to date are improved gait speed and reduced freezing of gait when associated with intensive physiotherapy.^4^ Identifying a neurophysiological effect could clarify whether TsMS engages mechanisms like those demonstrated in parkinsonian animals and support further clinical use and research.

This open‐label, pilot trial without placebo control assessed the acute effects of theta‐burst TsMS at the T3 spinal level on STN LFPs in two PD patients (Data S1) with bilateral STN DBS (Percept PC; Medtronic, Minneapolis, MN, USA). TsMS duration was 3 min and 58 s. All assessments occurred after a 12‐hr withdrawal from dopaminergic therapy and with DBS turned off.

Bilateral STN LFPs were recorded 6 min before and after TsMS, under two conditions: rest (3 min seated) and movement (1 min of repetitive hand opening/closing followed by 2 min of walking).

The LFP powers in the beta (13–35 Hz) and gamma (35–100 Hz) rhythms were analyzed to assess movement‐related desynchronization or synchronization (MRDS) for baseline and post‐TsMS conditions. MRDS is defined by the following equation:

$$\text{MRDS}x=\frac{\mathrm{PMx}-\mathrm{PRx}}{\mathrm{PRx}},$$

where *x* represents the rhythm (*β*: beta or *γ*: gamma), and PM_*X*_ and PR_*X*_ denote its power under movement and rest conditions, respectively^5^ (Data S1). This study was approved by the local ethics committee and all patients provided informed consent.

Power spectral density coefficients for rest and movement conditions before and after TsMS depicted a peak in the beta band power (Fig. 1). TsMS induced reduction of MRDS_*β*_ and an increase of P_R*γ*_ and MRDS*γ* (Fig. 1 and Table S1). As anticipated, no clinical changes occurred after one TsMS session.

**FIG. 1:**
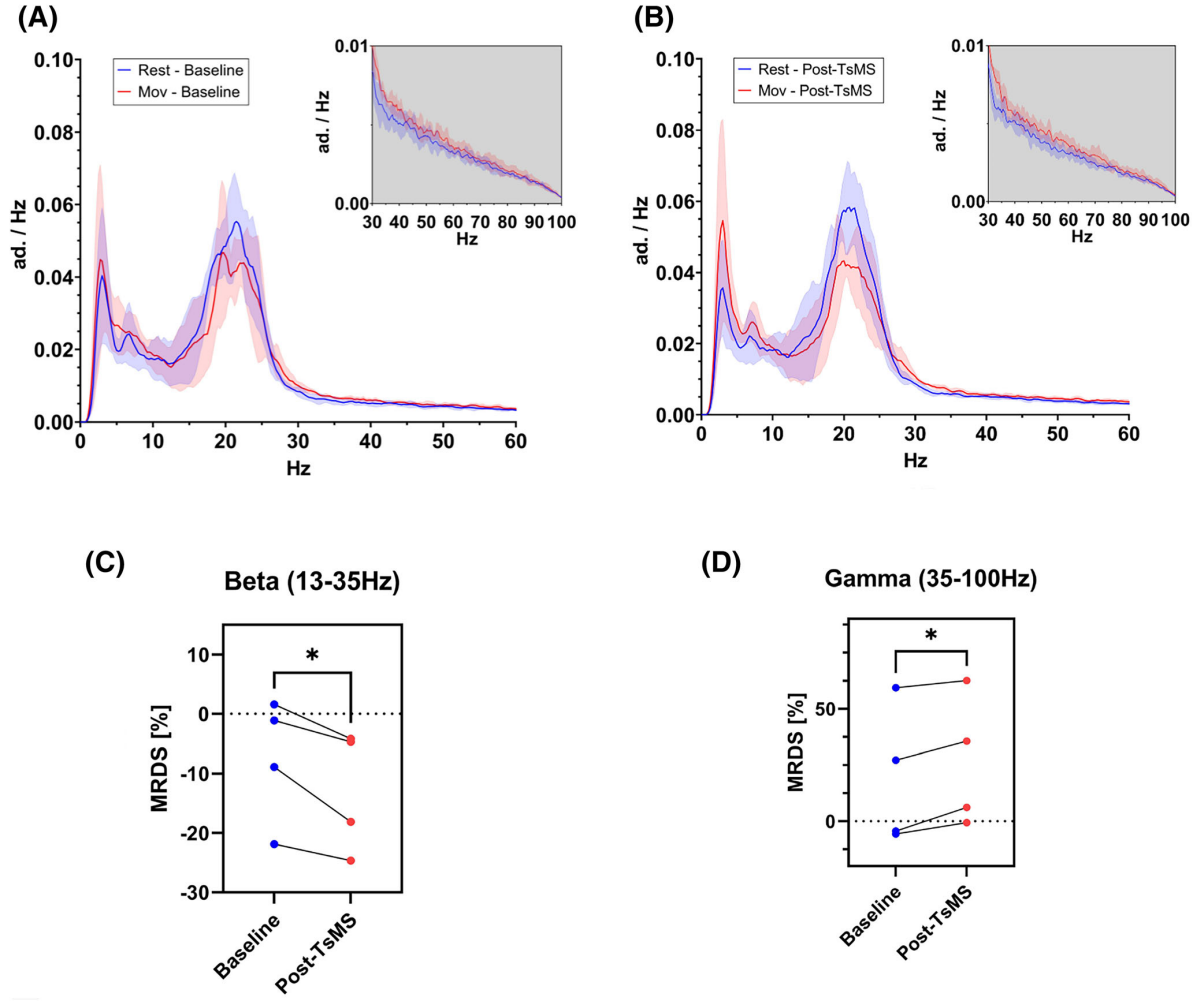
Mean power spectral density coefficients (continuous) and standard deviation (SD) (shaded) for the baseline (A) and after transcutaneous magnetic spinal cord stimulation (TsMS) (B) during rest (blue) and movement conditions (red). Paired beta movement‐related desynchronization or synchronization (MRDS) for the baseline (blue) and after stimulation (red) and descriptive statistics (mean ± SD). (C) Paired gamma MRDS for the baseline (blue) and after stimulation (red) and descriptive statistics (mean ± SD) (D). *P < 0.05. [Color figure can be viewed at wileyonlinelibrary.com]

TsMS can modulate STN electrophysiology in PD patients. Specifically, it reduced MRDS_*β*_ and increased MRDS_*γ*_, both biomarkers of a prokinetic state,^1^ suggesting that TsMS may promote favorable conditions to locomotion. These LFP changes support the hypothesis that TsMS engages ascending neural pathways to the mesencephalic locomotor area, disrupting abnormal low‐frequency corticostriatal oscillations and enhancing gamma synchrony, mimicking the effects of dopamine therapy or DBS.^1^ Similar findings have been reported with SCS.^6^

This single‐session trial with a small sample size did not produce significant changes in beta power, suggesting that multiple TsMS sessions may be necessary to enhance its effects and potentially improve PD symptoms. Another hypothesis is that SCS and TsMS efficacy may possibly depend on individualized parameter optimization.^7^ In conclusion, as questions remain about optimal TsMS stimulation paradigms, we hope these results encourage further LFP research and advance personalized, biomarker‐guided therapy development.

## Author Roles

(1) Research Project: A. Conception, B. Organization, C. Execution; (2) Statistical Analysis: A. Design, B. Execution, C. Review and Critique; (3) Manuscript: A. Writing of the First Draft, B. Review and Critique.

A.L.M.A.: 2C, 3A, 3B.

J.S.S.: 3A, 3B.

L.R.T.S.: 2A, 2B, 2C, 3B.

A.F.N.: 2A, 2B, 2C, 3B.

F.G.: 1A, 1B, 1C, 2C, 3B.

R.B.C.: 1A, 1B, 1C, 2A, 2B, 3A.

J.R.M.: 1B, 1C, 3B.

G.A.N.: 1B, 1C, 3B.

D.S.B.: 1B, 1C, 3B.

T.T.S.: 3B.

M.S.G.R.: 1A, 1B.

M.J.T.: 1B, 3B.

E.R.B.: 1B, 3B.

D.C.S.: 2A, 2B, 2C, 3B.

R.G.C.: 1A, 1B, 1C, 2C, 3B.

## Financial Disclosures of All Authors (for the Past 12 Months)

We would like to acknowledge the financial support of the Coordenação de Aperfeiçoamento de Pessoal de Nível Superior–Brasil (CAPES) (Finance Code 001). D.C.S. is funded by the National Council for Scientific and Technological Development (CNPq) (Grant No. 313970/2023‐8).

## Supporting information


**Data S1.** Supporting Information.

## Data Availability

The data that support the findings of this study are available from the corresponding author upon reasonable request.
