# Supplementary material for: Transcutaneous Spinal Magnetic Stimulation Affects Subthalamic Activity in Parkinson's Disease
Source: Mov Disord. 2025 Sep 3;40(11):2543–5. doi: 10.1002/mds.70035 (PMC12661619; doi:10.1002/mds.70035)
Supplement: Supplementary file 1 — Data S1. Supporting Information. [file MDS-40-2543-s001.docx]

**Supplementary File 1**

**Baseline patients’ characteristics**

|  | **Age** | **Gender** | **PD**  **duration** | **UPDRS III**  **(Off**  **medication)** | **DBS**  **indication** | **DBS therapy duration** |
| --- | --- | --- | --- | --- | --- | --- |
| Patient 1 | 55 | Female | 6 | 43 | Motor fluctuation | 2 |
| Patient 2 | 52 | Male | 6 | 67 | Motor fluctuation | 1 |

PD = Parkinson’s disease, UPDRS-III = Unified Parkinson’s Disease Rating Scale part III, STN DBS = Subthalamic nucleus deep brain stimulation.

**Stimulation parameters**

TsMS was delivered using a circular coil (Magventure R20) positioned perpendicular to the spinal cord at the level of the third thoracic vertebra, with intensity set to 100% of the motor threshold (eliciting thoracic nerve contractions). The upper thoracic level was selected due to its relatively thinner cross-sectional spinal cord area, which theoretically facilitates access to deeper structures. Moreover, this level has been employed in previous SCS studies and in preclinical investigations in monkeys (1–3). To ensure safety, the coil was placed over 10 cm away from any DBS system components (4,5). Stimulation consisted of intermittent theta-burst trains (20 bursts per train at 5 Hz) over 20 trains with 8-second intertrain intervals. Each burst contained three pulses at 50 Hz, totaling 1200 pulses in a single session with a total duration of 3 minutes and 58 seconds.

**Data extraction**

Data from the Percept™ PC system, recorded from both STNs (totaling four data points), were exported and processed offline using MATLAB (The MathWorks, Inc., Natick, Massachusetts, United States). Both patients' ventral contacts had the best LFP signal and clinical effect, so were selected for LFP recordings during the trial. 180s of subthalamic local field potentials were sampled at 250 Hz, bandpass (2 - 100 Hz) filtered through a sixth order IIR Butterworth filter and z-scored for each recording session. This normalization resulted in unitless, or adimensional (ad.), data values. The power spectral density (PSD) was estimated using Welch periodogram considering windows of 1000 samples (4 s) with overlap of 500 samples and 1000 samples for FFT evaluation, resulting in a spectral resolution of 0.25 Hz.

**Results**

To reduce the potential confounding effects of movement-related modulation on LFPs, power changes were computed using the following normalization formula: MRDSₓ = (PMₓ – PRₓ) / PRₓ, where PMₓ denotes the power during movement and PRₓ denotes the power at rest. This method was employed to normalize the data relative to baseline physiological fluctuations associated with voluntary motor activity.

**Table 1**. Mean ± standard deviation (SD) for beta and gamma power under rest (P_Rx_) and movement conditions (P_Mx_) and for beta and gamma movement-related desynchronization or synchronization (MRDS).

|  | Baseline | Post-TsMS | t(3); p-value |
| --- | --- | --- | --- |
| P_Rβ_ | 0.6119±0.0743 [ad] | 0.6323±0.0751 [ad] | 2.966; 0.0592 |
| P_Mβ_ | 0.5611±0.0492 [ad] | 0.5468±0.0565 [ad] | 2.660; 0.0764 |
| MRDS_β_ | -7.58±10.53 % | -12.92±10.14 % | 3.694; 0.0344 ***(*)*** |
| P_Rγ_ | 0.1810±0.0361 [ad] | 0.1682±0.0322 [ad] | 4.228; 0.0242 ***(*)*** |
| P_Mγ_ | 0.2081±0.0234 [ad] | 0.2059±0.0252 [ad] | 0.8711; 0.4478 |
| MRDS_γ_ | 19.12±30.81 % | 25.90±29.04 % | 4.089;0.0264 ***(*)*** |

β: beta; γ: gamma; * statistical significance (p < 0.05).

**References**

1. Fonoff ET, De Lima-Pardini AC, Coelho DB, Monaco BA, Machado B, Pinto De Souza C, et al. Spinal Cord Stimulation for Freezing of Gait: From Bench to Bedside. Front Neurol. 27 de agosto de 2019;10:905.

2. Benussi A, Batsikadze G, França C, Cury RG, Maas RPPWM. The Therapeutic Potential of Non-Invasive and Invasive Cerebellar Stimulation Techniques in Hereditary Ataxias. Cells. 20 de abril de 2023;12(8):1193.

3. Miguel A.L. Nicolelis MBS. Spinal Cord Stimulation Alleviates Motor Deficits in a Primate Model of Parkinson Disease. 19 de novembro de 2014;84:716–22.

4. Kumar R, Chen R, Ashby P. Safety of transcranial magnetic stimulation in patients with implanted deep brain stimulators. Mov Disord. janeiro de 1999;14(1):157–8.

5. Rossi S, Hallett M, Rossini PM, Pascual-Leone A. Safety, ethical considerations, and application guidelines for the use of transcranial magnetic stimulation in clinical practice and research. Clin Neurophysiol. dezembro de 2009;120(12):2008–39.
